# Supplementary material for: Arthropods of the great indoors: characterizing diversity inside urban and suburban homes
Source: PeerJ. 2016 Jan 19;4:e1582. doi: 10.7717/peerj.1582 (PMC4727974; doi:10.7717/peerj.1582)
Supplement: Table S1 [file peerj-04-1582-s001.docx]

| **Class** | **Order** | **Family** | ***Genus*** | ***species*** | **common name** | **% homes (n=50)** |
| --- | --- | --- | --- | --- | --- | --- |
| **Entognatha** |  |  |  |  | **non-insect hexapods** | **88** |
|  | **Collembola** |  |  |  | **springtails** | **88** |
|  |  | Entomobryidae |  |  | slender springtails | 78 |
|  |  | Hypogastruridae |  |  | hypogastrurid springtails | 6 |
|  |  | Isotomidae |  |  | isotomid springtails | 6 |
|  |  | Sminthuridae |  |  | globular springtails | 6 |
|  |  | Tomoceridae |  |  | elongate springtails | 26 |
| **Insecta** |  |  |  |  | **true insects** | **100** |
|  | **Archaeognatha** |  |  |  | **jumping bristletails** | **18** |
|  |  | Machilidae |  |  | jumping bristletails | 14 |
|  |  | Meinertellidae |  |  | rock bristletails | 4 |
|  | **Zygentoma** |  |  |  | **silverfish** | **68** |
|  |  | Lepismatidae |  |  | silverfish | 68 |
|  |  |  | *Ctenolepisma* |  | silverfish |  |
|  |  |  | *Lepisma* | *saccharina* | common silverfish |  |
|  | **Odonata** |  |  |  | **dragonflies & damselflies** | **2** |
|  |  | Coenagrionidae |  |  | narrow-winged damselflies | 2 |
|  | **Ephemeroptera** |  |  |  | **mayflies** | **2** |
|  |  | Heptageniidae |  |  | flatheaded mayflies | 2 |
|  | **Plecoptera** |  |  |  | **stoneflies** | **2** |
|  |  | Leuctridae |  |  | rolled-winged stoneflies | 2 |
|  | **Dermaptera** |  |  |  | **earwigs** | **50** |
|  |  | Anisolabididae |  |  | earwigs | 26 |
|  |  |  | *Euborellia* |  | earwigs |  |
|  |  |  | *Euborellia* | *annulipes* | ring-legged earwig |  |
|  |  | Forficulidae |  |  | earwigs | 24 |
|  |  |  | *Forficula* | *auricularia* | European earwig |  |
|  |  | Spongiphoridae |  |  | little earwigs | 4 |
|  |  |  | *Labia* | *minor* | lesser earwig |  |
|  | **Orthoptera** |  |  |  | **grasshoppers, crickets & katydids** | **76** |
|  |  | Acrididae |  |  | short horned grasshoppers | 2 |
|  |  | Gryllidae |  |  | crickets | 30 |
|  |  |  | *Anaxipha* |  | brown trigs |  |
|  |  |  | *Cyrtoxipha* |  | trigs |  |
|  |  | Mogoplistidae |  |  | scaly crickets | 6 |
|  |  |  | *Cycloptilum* |  | scaly crickets |  |
|  |  | Myrmecophilidae |  |  | ant-loving crickets | 10 |
|  |  |  | *Myrmecophilus* | *pergandei* | Eastern ant cricket |  |
|  |  | Rhaphidophoridae |  |  | camel & cave crickets | 58 |
|  |  |  | *Ceuthophilus* |  | camel crickets |  |
|  |  |  | *Diestrammena* |  | camel crickets |  |
|  |  | Tettigoniidae |  |  | katydids | 4 |
|  |  |  | *Atlanticus* |  | Eastern shieldbacks |  |
|  | **Blattodea** |  |  |  | **cockroaches** | **82** |
|  |  | Blattidae |  |  | cockroaches | 74 |
|  |  |  | *Periplaneta* |  | cockroaches |  |
|  |  |  | *Periplaneta* | *americana* | American cockroach |  |
|  |  |  | *Periplaneta* | *australasiae* | Australian cockroach |  |
|  |  |  | *Periplaneta* | *fuliginosa* | smoky brown cockroach |  |
|  |  | Ectobiidae |  |  | cockroaches | 34 |
|  |  |  | *Blattella* |  | cockroaches |  |
|  |  |  | *Blattella* | *germanica* | German cockroach |  |
|  |  |  | *Cariblatta* | *lutea* | small yellow cockroach |  |
|  |  |  | *Parcoblatta* |  | woodroaches |  |
|  | **Isoptera** |  |  |  | **termites** | **28** |
|  |  | Rhinotermitidae |  |  | subterranean termites | 28 |
|  |  |  | *Reticulitermes* |  | subterranean termites |  |
|  |  |  | *Reticulitermes* | *flavipes* | Eastern subterranean termite |  |
|  |  |  | *Reticulitermes* | *virginicus* | Southeastern subterranean termite |  |
|  | **Hemiptera** |  |  |  | **true bugs** | **98** |
|  |  | Acanaloniidae |  |  | acanaloniid planthoppers | 4 |
|  |  |  | *Acanalonia* |  | acanaloniid planthoppers |  |
|  |  | Aleyrodidae |  |  | whiteflies | 2 |
|  |  | Alydidae |  |  | broad-headed bugs | 2 |
|  |  | Anthocoridae |  |  | minute pirate bugs | 41 |
|  |  |  | *Amphiareus* |  | minute pirate bugs |  |
|  |  | Aphididae |  |  | aphids | 56 |
|  |  | Aradidae |  |  | flat bugs | 4 |
|  |  | Berytidae |  |  | stilt bugs | 2 |
|  |  | Blissidae |  |  | chinch bugs | 2 |
|  |  |  | *Blissus* |  | chinch bugs |  |
|  |  | Cercopidae |  |  | froghoppers & spittle bugs | 6 |
|  |  |  | *Aphrophora* |  | spittle bugs |  |
|  |  |  | *Prosapia* |  | spittle bugs |  |
|  |  |  | *Prosapia* | *bicincta* | two-lined spittle bug |  |
|  |  | Cicadellidae |  |  | leafhoppers | 82 |
|  |  | Cicadidae |  |  | cicadas | 2 |
|  |  |  | *Magicicada* |  | periodical cicadas |  |
|  |  | Cimicidae |  |  | bed bugs, bat bugs, swallow bugs | 2 |
|  |  |  | *Cimex* | *adjunctus* | Eastern bat bug |  |
|  |  | Cixiidae |  |  | cixiid planthoppers | 8 |
|  |  |  | *Pintalia* |  | cixiid planthoppers |  |
|  |  | Coreidae |  |  | squash & leaf-footed bugs | 12 |
|  |  |  | *Leptoglossus* |  | leaf-footed bugs |  |
|  |  | Cydnidae |  |  | burrower bugs | 6 |
|  |  |  | *Amnestus* |  | burrower bugs |  |
|  |  | Delphacidae |  |  | delphacid planthoppers | 12 |
|  |  | Derbidae |  |  | derbid planthoppers | 6 |
|  |  |  | *Cedusa* |  | derbid planthoppers |  |
|  |  |  | *Cedusa* | *maculata* | derbid planthopper |  |
|  |  | Dictyopharidae |  |  | dictyopharid planthoppers | 2 |
|  |  | Flatidae |  |  | flatid planthoppers | 4 |
|  |  |  | *Metcalfa* | *pruinosa* | citrus flatid |  |
|  |  | Fulgoridae |  |  | lanternflies | 2 |
|  |  | Geocoridae |  |  | big-eyed bugs | 2 |
|  |  | Issidae |  |  | issid planthoppers | 2 |
|  |  |  | *Thionia* |  | issid planthoppers |  |
|  |  | Lasiochilidae |  |  | lasiochilid bugs | 4 |
|  |  | Lygaeidae |  |  | seed bugs | 10 |
|  |  |  | *Kleidocerys* | *resedae* | birch catkin bug |  |
|  |  | Membracidae |  |  | treehoppers | 2 |
|  |  | Miridae |  |  | plant bugs | 44 |
|  |  |  | *Halticus* |  | fleahoppers |  |
|  |  |  | *Rhinocapsus* |  | plant bugs |  |
|  |  |  | *Rhinocapsus* | *vanduzeei* | azalea plant bug |  |
|  |  | Pentatomidae |  |  | stink bugs | 22 |
|  |  |  | *Banasa* | *euchlora* | juniper stink bug |  |
|  |  |  | *Brochymena* |  | rough stink bugs |  |
|  |  |  | *Halyomorpha* | *halys* | brown marmorated stink bug |  |
|  |  | Phylloxeridae |  |  | phylloxerans | 4 |
|  |  | Psyllidae s.l. |  |  | jumping plant lice | 10 |
|  |  |  | *Livia* |  | jumping plant lice |  |
|  |  | Reduviidae |  |  | assassin bugs | 28 |
|  |  |  | *Empicoris* |  | thread-legged bugs |  |
|  |  |  | *Sinea* |  | spined assassin bugs |  |
|  |  |  | *Zelus* |  | assassin bugs |  |
|  |  |  | *Pselliopus* |  | assassin bugs |  |
|  |  | Rhyparochromidae |  |  | dirt-colored seed bugs | 30 |
|  |  |  | *Myodocha* |  | long-necked seed bugs |  |
|  |  |  | *Ptochiomera* | *nodosa* | dirt-colored seed bug |  |
|  |  | Tingidae |  |  | lace bugs | 16 |
|  |  |  | *Corythucha* |  | lace bugs |  |
|  | **Psocodea** |  |  |  | **lice** | **98** |
|  |  | Amphipsocidae |  |  | amphipsocid bark lice | 2 |
|  |  |  | *Polypsocus* | *corruptus* | bark louse |  |
|  |  | Caeciliusidae |  |  | caeciliusid bark lice | 2 |
|  |  | Ectopsocidae |  |  | ectopsocid bark lice | 16 |
|  |  | Hemipsocidae |  |  | hemipsocid bark lice | 2 |
|  |  | Lepidopsocidae |  |  | scaly-winged bark lice | 24 |
|  |  | Liposcelididae |  |  | book lice | 98 |
|  |  |  | *Liposcelis* |  | book lice |  |
|  |  | Mesopsocidae |  |  | mesopsocid bark lice | 2 |
|  |  | Myopsocidae |  |  | myopsocid bark lice | 2 |
|  |  | Psoquillidae |  |  | psoquillid bark lice | 2 |
|  |  | Psyllopsocidae |  |  | psyllopsocid bark lice | 2 |
|  |  | Stenopsocidae |  |  | stenopsocid bark lice | 8 |
|  |  |  | *Graphopsocus* | *cruciatus* | stenopsocid bark louse |  |
|  | **Thysanoptera** |  |  |  | **thrips** | **50** |
|  |  | Phlaeothripidae |  |  | tube-tailed thrips | 14 |
|  |  | Thripidae |  |  | common thrips | 32 |
|  | **Hymenoptera** |  |  |  | **wasps, ants & bees** | **100** |
|  |  | Aphelinidae |  |  | aphelinid wasps | 4 |
|  |  | Apidae |  |  | bees | 6 |
|  |  |  | *Apis* | *mellifera* | European honey bee |  |
|  |  |  | *Bombus* |  | bumble bees |  |
|  |  |  | *Xylocopa* |  | carpenter bees |  |
|  |  | Bethylidae |  |  | bethylid wasps | 28 |
|  |  | Braconidae |  |  | braconid wasps | 52 |
|  |  | Ceraphronidae |  |  | ceraphronid wasps | 14 |
|  |  | Chalcididae |  |  | chalcidid wasps | 14 |
|  |  | Chrysididae |  |  | cuckoo wasps | 6 |
|  |  | Cynipidae |  |  | gall wasps | 6 |
|  |  | Diapriidae |  |  | diapriid wasps | 26 |
|  |  |  | *Basalys* |  | diapriid wasps |  |
|  |  |  | *Coptera* |  | diapriid wasps |  |
|  |  | Encyrtidae |  |  | encyrtid wasps | 18 |
|  |  | Eulophidae |  |  | eulophid wasps | 70 |
|  |  |  | *Aprostocetus* |  | eulophid wasps |  |
|  |  |  | *A. (Tetrastichodes)* | *hagenowii* | eulophid wasps |  |
|  |  | Eupelmidae |  |  | eupelmid wasps | 2 |
|  |  | Eurytomidae |  |  | eurytomid wasps | 6 |
|  |  | Figitidae |  |  | figitid wasps | 4 |
|  |  | Formicidae |  |  | ants | 100 |
|  |  |  | *Aphaenogaster* |  | winnow ants |  |
|  |  |  | *Brachyponera* | *chinensis* | Asian needle ant |  |
|  |  |  | *Camponotus* |  | carpenter ants |  |
|  |  |  | *Camp. (Colobopsis)* |  | carpenter ants |  |
|  |  |  | *Camponotus* | *nearcticus* | carpenter ant |  |
|  |  |  | *Camponotus* | *pennsylvanicus* | Eastern black carpenter ant |  |
|  |  |  | *Crematogaster* |  | acrobat ants |  |
|  |  |  | *Formica* |  | wood & mound ants |  |
|  |  |  | *Hypoponera* |  | ponerine ants |  |
|  |  |  | *Lasius* |  | cornfield & citronella ants |  |
|  |  |  | *Linepithema* | *humile* | Argentine ant |  |
|  |  |  | *Monomorium* |  | little ants |  |
|  |  |  | *Monomorium* | *minimum* | little black ant |  |
|  |  |  | *Nylanderia* |  | crazy ants |  |
|  |  |  | *Pheidole* |  | big-headed ants |  |
|  |  |  | *Pogonomyrmex* |  | harvester ants |  |
|  |  |  | *Prenolepsis* | *imparis* | winter ant |  |
|  |  |  | *Proceratium* | *silaceum* | proceratiine ant |  |
|  |  |  | *Solenopsis* |  | fire & thief ants |  |
|  |  |  | *Solenopsis* | *invicta* | red imported fire ant |  |
|  |  |  | *Stigmatomma* |  | Dracula ants |  |
|  |  |  | *Strumigenys* |  | miniature trap-jaw ants |  |
|  |  |  | *Tapinoma* |  | odorous & ghost ants |  |
|  |  |  | *Tapinoma* | *sessile* | odorous house ant |  |
|  |  |  | *Temnothorax* |  | acorn ants |  |
|  |  |  | *Tetramorium* |  | pavement ants |  |
|  |  | Halictidae |  |  | sweat bees | 10 |
|  |  | Ichneumonidae |  |  | ichneumon wasps | 38 |
|  |  |  | *Enicospilus* |  | ichneumon wasps |  |
|  |  | Megachilidae |  |  | leaf-cutter & mason bees | 6 |
|  |  |  | *Osmia* |  | mason bees |  |
|  |  | Megaspilidae |  |  | megaspilid wasps | 4 |
|  |  | Mutillidae |  |  | velvet ants | 6 |
|  |  |  | *Dasymutilla* |  | velvet ants |  |
|  |  |  | *Pseudomethoca* |  | velvet ants |  |
|  |  | Mymaridae |  |  | fairyflies | 26 |
|  |  | Perilampidae |  |  | perilampid wasps | 2 |
|  |  | Platygastridae s.l. |  |  | platygastrid wasps | 60 |
|  |  | Pompilidae |  |  | spider wasps | 34 |
|  |  |  | *Tachypompilus* |  | spider wasps |  |
|  |  | Proctotrupidae |  |  | proctotrupid wasps | 4 |
|  |  | Pteromalidae |  |  | pteromalid wasps | 42 |
|  |  | Rhopalosomatidae |  |  | rhopalosomatid wasps | 2 |
|  |  |  | *Rhopalosoma* | *nearcticum* | rhopalosomatid wasp |  |
|  |  | Sphecidae s.l. |  |  | hunting wasps | 26 |
|  |  |  | *Chlorion* |  | steel-blue hunting wasps |  |
|  |  |  | *Sceliphron* |  | mud daubers |  |
|  |  | Tiphiidae |  |  | tiphiid wasps | 2 |
|  |  | Torymidae |  |  | torymid wasps | 8 |
|  |  | Trichogrammatidae |  |  | trichogrammatid wasps | 2 |
|  |  | Vespidae |  |  | hornets, potter & paper wasps | 14 |
|  |  |  | *Polistes* |  | paper wasps |  |
|  |  | Xyelidae |  |  | xyelid sawflies | 2 |
|  |  |  | *Xyela* |  | xyelid sawflies |  |
|  | **Neuroptera** |  |  |  | **lacewings, antlions, etc.** | **56** |
|  |  | Berothidae |  |  | beaded lacewings | 2 |
|  |  |  | *Lomomyia* |  | beaded lacewings |  |
|  |  | Chrysopidae |  |  | green lacewings | 34 |
|  |  | Coniopterygidae |  |  | dustywings | 16 |
|  |  | Hemerobiidae |  |  | brown lacewings | 18 |
|  |  | Myrmeleontidae |  |  | antlions | 4 |
|  |  |  | *Dendroleon* | *obsoletus* | antlion |  |
|  | **Coleoptera** |  |  |  | **beetles** | **100** |
|  |  | Aderidae |  |  | ant-like leaf beetles | 22 |
|  |  | Anobiidae |  |  | death watch & spider beetles | 60 |
|  |  |  | *Caenocara* |  | puffball beetle |  |
|  |  |  | *Lasioderma* |  | cigarette beetles |  |
|  |  |  | *Lasioderma* | *serricorne* | cigarette beetle |  |
|  |  |  | *Ptinus* |  | woodworm/wood borer |  |
|  |  |  | *Stegobium* | *panaceum* | drugstore beetle |  |
|  |  |  | *Trichodesma* |  | death watch beetles |  |
|  |  | Anthicidae |  |  | ant-like flower beetles | 18 |
|  |  |  | *Notoxus* |  | ant-like flower beetles |  |
|  |  | Anthribidae |  |  | fungus weevils | 8 |
|  |  | Biphyllidae |  |  | false skin beetles | 2 |
|  |  |  | *Diplocaulus* |  | false skin beetles |  |
|  |  | Bostrichidae |  |  | horned powder post beetles | 2 |
|  |  | Buprestidae |  |  | metallic wood boring beetles | 2 |
|  |  |  | *Buprestes* | *rufipes* | red-legged buprestis |  |
|  |  | Cantharidae |  |  | soldier beetles | 8 |
|  |  |  | *Atalantycha* |  | soldier beetles |  |
|  |  |  | *Ditemnus* |  | soldier beetles |  |
|  |  |  | *Podabrus* |  | soldier beetles |  |
|  |  | Carabidae |  |  | ground beetles | 66 |
|  |  |  | *Galerita* |  | false bombardier beetles |  |
|  |  |  | *Galerita* | *bicolor* | false bombardier beetle |  |
|  |  |  | *Scarites* |  | pedunculate ground beetle |  |
|  |  |  | *Notiophilus* |  | ground beetles |  |
|  |  | Cerambycidae |  |  | longhorned beetles | 16 |
|  |  |  | *Anelaphus* |  | longhorned beetles |  |
|  |  |  | *Euderces* |  | longhorned beetles |  |
|  |  |  | *Euderces* | *pini* | longhorned beetle |  |
|  |  |  | *Urgleptes* |  | longhorned beetles |  |
|  |  |  | *Urgleptes* | *facetus* | longhorned beetle |  |
|  |  | Chrysomelidae |  |  | leaf beetles | 46 |
|  |  |  | *Disonycha* | *triangularis* | leaf beetle |  |
|  |  |  | *Glyptoscelis* |  | leaf beetles |  |
|  |  |  | *Glyptoscelis* | *pubescens* | leaf beetle |  |
|  |  | Clambidae |  |  | minute beetles | 2 |
|  |  | Cleridae |  |  | checkered beetles | 18 |
|  |  |  | *Cymatodera* |  | checkered beetles |  |
|  |  | Coccinellidae |  |  | ladybugs | 52 |
|  |  |  | *Harmonia* | *axyridis* | multicolored Asian lady beetle |  |
|  |  | Corylophidae |  |  | minute hooded beetles | 8 |
|  |  | Cryptophagidae |  |  | silken fungus beetles | 26 |
|  |  |  | *Caenoscelis* |  | silken fungus beetles |  |
|  |  | Cupedidae |  |  | reticulated beetles | 2 |
|  |  |  | *Tenomerga* | *cinerea* | reticulated beetle |  |
|  |  | Curculionidae |  |  | weevils | 82 |
|  |  |  | *Curculio* |  | weevils |  |
|  |  |  | *Sitophilus* |  | granary weevils |  |
|  |  |  | *Stenoscelis* |  | weevils |  |
|  |  |  | *Xyleborinus* | *saxesenii* | fruit-tree borer |  |
|  |  | Dermestidae |  |  | carpet & larder beetles | 100 |
|  |  |  | *Anthrenus* |  | carpet beetles |  |
|  |  |  | *Anthrenus* | *verbasci* | varied carpet beetle |  |
|  |  |  | *Apsectus* |  | dermestid beetle |  |
|  |  |  | *Attagenus* |  | black carpet beetles |  |
|  |  |  | *Dermestes* |  | larder beetles |  |
|  |  |  | *Novelsis* |  | dermestid beetles |  |
|  |  |  | *Trogoderma* |  | warehouse & cabinet beetles |  |
|  |  | Elateridae |  |  | click beetles | 74 |
|  |  |  | *Conoderus* |  | click beetles |  |
|  |  |  | *Conoderus* | *pictus* | click beetle |  |
|  |  |  | *Lacon* |  | click beetles |  |
|  |  | Endomychidae |  |  | handsome fungus beetles | 6 |
|  |  |  | *Holoparamecus* |  | handsome fungus beetle |  |
|  |  | Erotylidae |  |  | pleasing fungus beetles | 6 |
|  |  |  | *Cryptophilus* |  | pleasing fungus beetles |  |
|  |  | Eucinetidae |  |  | plate thigh beetles | 2 |
|  |  | Eucnemidae |  |  | false click beetles | 4 |
|  |  | Geotrupidae |  |  | earth-boring dung beetles | 2 |
|  |  | Heteroceridae |  |  | variegated mud-loving beetles | 2 |
|  |  | Histeridae |  |  | clown beetles | 10 |
|  |  | Hydrophilidae |  |  | water scavenger beetles | 2 |
|  |  | Laemophloeidae |  |  | lined flat bark beetles | 6 |
|  |  |  | *Placonotus* |  | lined flat bark beetles |  |
|  |  | Lampyridae |  |  | fireflies | 20 |
|  |  |  | *Lucidota* |  | diurnal fireflies |  |
|  |  |  | *Lucidota* | *atra* | black firefly |  |
|  |  | Latridiidae |  |  | minute brown scavenger beetles | 38 |
|  |  |  | *Cartodere* |  | minute brown scavenger beetles |  |
|  |  |  | *Cartodere* | *constricta* | plaster beetle |  |
|  |  |  | *Eufallia* | *seminiveus* |  |  |
|  |  | Leiodidae |  |  | round fungus beetles | 6 |
|  |  |  | *Agathidium* |  | round fungus beetles |  |
|  |  | Lucanidae |  |  | stag beetles | 4 |
|  |  |  | *Lucanus* |  | stag beetles |  |
|  |  |  | *Lucanus* | *elaphus* | giant stag beetle |  |
|  |  | Lycidae |  |  | net-winged beetles | 4 |
|  |  | Melandryidae |  |  | false darkling beetles | 2 |
|  |  |  | *Microtonus* | *sericans* | false darkling beetle |  |
|  |  | Melyridae |  |  | soft-winged flower beetles | 20 |
|  |  |  | *Anthocomus* | *equestris* | soft-winged flower beetle |  |
|  |  |  | *Attalus* |  | soft-winged flower beetles |  |
|  |  |  | *Attalus* | *scincetus* | soft-winged flower beetle |  |
|  |  | Micromalthidae |  |  | telephone-pole beetles | 2 |
|  |  |  | *Micromalthus* | *debilis* | telephone-pole beetle |  |
|  |  | Monotomidae |  |  | root-eating beetles | 6 |
|  |  |  | *Monotoma* |  | root-eating beetles |  |
|  |  | Mordellidae |  |  | tumbling flower beetles | 24 |
|  |  | Mycetophagidae |  |  | hairy fungus beetles | 20 |
|  |  |  | *Litargus* |  | hairy fungus beetles |  |
|  |  |  | *Litargus* | *sexpunctatus* | hairy fungus beetle |  |
|  |  | Nitidulidae |  |  | sap beetles | 24 |
|  |  |  | *Carpophilus* |  | sap beetles |  |
|  |  |  | *Conotelus* |  | sap beetles |  |
|  |  |  | *Lobiopa* |  | sap beetles |  |
|  |  |  | *Pallodes* |  | sap beetles |  |
|  |  |  | *Stelidota* |  | sap beetles |  |
|  |  |  | *Stelidota* | *geminata* | strawberry sap beetle |  |
|  |  | Passalidae |  |  | bess beetles | 2 |
|  |  |  | *Odontotaenius* | *disjunctus* | bess beetle |  |
|  |  | Phalacridae |  |  | shining flower beetles | 12 |
|  |  | Ptiliidae |  |  | feather-winged beetles | 6 |
|  |  | Ptilodactylidae |  |  | ptilodactylid beetles | 30 |
|  |  |  | *Ptilodactyla* |  | ptilodactylid beetles |  |
|  |  | Scarabaeidae |  |  | scarab beetles | 52 |
|  |  |  | *Aphodius* |  | aphodiine dung beetles |  |
|  |  |  | *Aphodius* | *granarius* | aphodiine dung beetle |  |
|  |  |  | *Ataenius* |  | aphodiine dung beetles |  |
|  |  |  | *Ataenius* | *imbricatus* | aphodiine dung beetle |  |
|  |  |  | *Callistethus* | *marginatus* | shining leaf chafer |  |
|  |  |  | *Cyclocephala* |  | masked chafers |  |
|  |  |  | *Cyclocephala* | *borealis* | Northern masked chafer |  |
|  |  |  | *Euphoria* |  | flower chafers |  |
|  |  |  | *Euphoria* | *sepulcralis* | dark flower scarab |  |
|  |  |  | *Euetheola* | *humilis* | sugarcane beetle |  |
|  |  |  | *Exomala* | *orientalis* | Oriental beetle |  |
|  |  |  | *Hoplia* |  | monkey beetles |  |
|  |  |  | *Onthophagus* |  | dung beetles |  |
|  |  |  | *Onthophagus* | *pennsylvanicus* | dung beetle |  |
|  |  |  | *Pelidnota* | *punctata* | grapevine beetle |  |
|  |  |  | *Serica* |  | May beetles |  |
|  |  | Scirtidae |  |  | marsh beetles | 4 |
|  |  |  | *Cyphon* |  | marsh beetles |  |
|  |  |  | *Sacodes* |  | marsh beetles |  |
|  |  | Scraptiidae |  |  | false flower beetles | 20 |
|  |  | Scydmaenidae |  |  | ant-like stone beetles | 4 |
|  |  | Silvanidae |  |  | silvanid flat bark beetles | 46 |
|  |  |  | *Ahasverus* |  | silvanid flat bark beetles |  |
|  |  |  | *Oryzaephilus* |  | grain beetles |  |
|  |  |  | *Oryzaephilus* | *surinamensis* | sawtooth grain beetle |  |
|  |  | Staphylinidae |  |  | rove beetles | 54 |
|  |  |  | *Stenus* |  | water skaters |  |
|  |  | Tenebrionidae |  |  | darkling beetles | 62 |
|  |  |  | *Anaedus* |  | darkling beetles |  |
|  |  |  | *Neomida* |  | darkling beetles |  |
|  |  |  | *Platydema* |  | darkling beetles |  |
|  |  |  | *Platydema* | *cyanescens* | darkling beetle |  |
|  |  |  | *Statira* |  | darkling beetles |  |
|  |  |  | *Tribolium* |  | flour beetles |  |
|  |  |  | *Uloma* |  | darkling beetles |  |
|  |  | Tetratomidae |  |  | polypore fungus beetles | 2 |
|  |  |  | *Holostrophus* | *bifasciatus* | polypore fungus beetle |  |
|  |  | Throscidae |  |  | false metallic wood | 22 |
|  |  | Trogidae |  |  | hide beetles | 2 |
|  |  | Trogossitidae |  |  | bark-gnawing beetles | 16 |
|  |  |  | *Corticotomus* |  | bark-gnawing beetles |  |
|  |  |  | *Tenebroides* |  | bark-gnawing beetles |  |
|  |  | Zopheridae |  |  | ironclad beetles | 16 |
|  |  |  | *Aulonium* |  | ironclad beetles |  |
|  |  |  | *Bitoma* |  | ironclad beetles |  |
|  |  |  | *Bitoma* | *quadriguttata* | ironclad beetle |  |
|  |  |  | *Colydium* |  | ironclad beetles |  |
|  |  |  | *Hyporhagus* |  | ironclad beetles |  |
|  |  |  | *Pycnomerus* |  | ironclad beetles |  |
|  |  |  | *Pycnomerus* | *sulcicollis* | ironclad beetle |  |
|  | **Lepidoptera** |  |  |  | **moths and butterflies** | **92** |
|  |  | Erebidae |  |  | owlet moths | 2 |
|  |  | Gelechiidae |  |  | gelechiid moths | 2 |
|  |  | Geometridae |  |  | geometer oths | 12 |
|  |  | Lymantriidae |  |  | tussock moths | 2 |
|  |  | Noctuidae |  |  | owlet moths | 44 |
|  |  | Pieridae |  |  | sulphur butterflies | 2 |
|  |  | Pyralidae |  |  | snout moths | 62 |
|  |  |  | *Plodia* | *interpunctella* | Indian meal moth |  |
|  |  | Tineidae |  |  | clothes moths | 60 |
|  |  | Tortricidae |  |  | leafroller moths | 10 |
|  |  | Yponomeutidae |  |  | ermine moths | 2 |
|  |  |  | *Atteva* | *aurea* | ailanthus webworm moth |  |
|  | **Trichoptera** |  |  |  | **caddisflies** | **12** |
|  |  | Hydropsychidae |  |  | netspinning caddisflies | 8 |
|  | **Siphonaptera** |  |  |  | **fleas** | **10** |
|  |  | Pulicidae |  |  | cat, dog & human fleas | 10 |
|  |  |  | *Ctenocephalides* |  | cat & dog fleas |  |
|  |  |  | *Ctenocephalides* | *felis* | cat flea |  |
|  | **Diptera** |  |  |  | **true flies** | **100** |
|  |  | Agromyzidae |  |  | leafminer flies | 12 |
|  |  | Anisopodidae |  |  | wood gnats | 10 |
|  |  |  | *Mycetobia* | *divergens* | wood gnat |  |
|  |  |  | *Sylvicola* |  | wood gnats |  |
|  |  | Anthomyiidae |  |  | root maggot flies | 10 |
|  |  | Asilidae |  |  | robber flies | 2 |
|  |  |  | *Ommatius* |  | robber flies |  |
|  |  | Bibionidae |  |  | March flies | 26 |
|  |  |  | *Bibio* |  | March flies |  |
|  |  |  | *Dilophus* |  | March flies |  |
|  |  | Calliphoridae |  |  | blow flies | 48 |
|  |  |  | *Calliphora* |  | blow flies |  |
|  |  |  | *Lucilia* |  | green bottle flies |  |
|  |  |  | *Lucilia* | *sericata* | common green bottle fly |  |
|  |  |  | *Pollenia* |  | cluster flies |  |
|  |  | Carnidae |  |  | carnid flies | 2 |
|  |  | Cecidomyiidae |  |  | gall midges | 100 |
|  |  | Ceratopogonidae |  |  | biting midges | 54 |
|  |  | Chaoboridae |  |  | phantom midges | 14 |
|  |  | Chironomidae |  |  | non-biting midges | 80 |
|  |  |  | *Ablabesmyia* |  | non-biting midges |  |
|  |  | Chloropidae |  |  | frit flies | 28 |
|  |  |  | *Ectacephala* |  | frit flies |  |
|  |  | Chyromyidae |  |  | chyromyid flies | 2 |
|  |  |  | *Gymnochiromyia* |  | chyromyid flies |  |
|  |  | Culicidae |  |  | mosquitoes | 82 |
|  |  |  | *Aedes* |  | mosquitoes |  |
|  |  |  | *Aedes* | *albopictus* | Asian tiger mosquito |  |
|  |  |  | *Anopheles* |  | malaria mosquitoes |  |
|  |  |  | *Anopheles* | *punctipennis* | malaria mosquito |  |
|  |  |  | *Culex* |  | house mosquitoes |  |
|  |  | Dolichopodidae |  |  | longlegged flies | 44 |
|  |  |  | *Condylostylus* |  | longlegged flies |  |
|  |  | Drosophilidae |  |  | fruit or vinegar flies | 66 |
|  |  |  | *Drosophila* |  | fruit or vinegar flies |  |
|  |  |  | *Drosophila* | *suzukii* | spotted wing drosophila |  |
|  |  |  | *Chymomyza* |  | fruit or vinegar flies |  |
|  |  |  | *Leucophenga* |  | fruit or vinegar flies |  |
|  |  |  | *Scaptomyza* |  | fruit or vinegar flies |  |
|  |  | Empididae s.l. |  |  | dance flies | 18 |
|  |  |  | *Hilara* |  | balloon flies |  |
|  |  |  | *Rhamphomyia* |  | dance flies |  |
|  |  | Ephydridae |  |  | shore flies | 14 |
|  |  |  | *Ochthera* |  | mantis flies |  |
|  |  |  | *Leptopsilopa* |  | shore flies |  |
|  |  | Fanniidae |  |  | lesser house flies | 10 |
|  |  |  | *Fannia* |  | lesser house flies |  |
|  |  | Heleomyzidae |  |  | heleomyzid flies | 2 |
|  |  | Lauxaniidae |  |  | lauxaniid flies | 16 |
|  |  |  | *Homoneura* |  | lauxaniid flies |  |
|  |  | Lonchopteridae |  |  | spear-winged flies | 2 |
|  |  |  | *Lonchoptera* |  | spear-winged flies |  |
|  |  | Milichiidae |  |  | freeloader flies | 14 |
|  |  |  | *Desmometopa* |  | freeloader flies |  |
|  |  |  | *Desmometopa* | *m-nigrum* | freeloader fly |  |
|  |  | Muscidae |  |  | house & stable flies | 44 |
|  |  |  | *Atherigona* |  | shoot flies |  |
|  |  |  | *Coenosia* |  | tiger flies |  |
|  |  |  | *Hydrotaea* |  | garbage flies |  |
|  |  |  | *Musca* |  | house & face flies |  |
|  |  |  | *Musca* | *domestica* | house fly |  |
|  |  | Mycetophilidae s.l. |  |  | fungus gnats | 68 |
|  |  |  | *Cordyla* |  | fungus gnats |  |
|  |  |  | *Keroplatus* |  | predatory fungus gnats |  |
|  |  |  | *Mycetophila* |  | fungus gnats |  |
|  |  | Phoridae |  |  | scuttle flies | 82 |
|  |  |  | *Dohrniphora* |  | scuttle flies |  |
|  |  | Pipunculidae |  |  | big-headed flies | 2 |
|  |  | Psychodidae |  |  | moth & drain flies | 74 |
|  |  |  | *Clogmia* | *albipunctata* | drain fly |  |
|  |  |  | *Psychoda* |  | moth flies |  |
|  |  | Rhinophoridae |  |  | woodlouse flies | 2 |
|  |  |  | *Melanophora* | *roralis* | woodlouse fly |  |
|  |  | Sarcophagidae |  |  | flesh flies | 38 |
|  |  | Scatopsidae |  |  | minute black scavenger flies | 50 |
|  |  | Scenopinidae |  |  | window flies | 2 |
|  |  | Sciaridae |  |  | dark-winged fungus gnats | 96 |
|  |  |  | *Sciara* |  | dark-winged fungus gnats |  |
|  |  | Sepsidae |  |  | black scavenger flies | 2 |
|  |  | Sphaeroceridae |  |  | lesser dung flies | 28 |
|  |  |  | *Parasphaerocera* |  | lesser dung flies |  |
|  |  | Stratiomyidae |  |  | soldier flies | 22 |
|  |  |  | *Hermetia* |  | soldier flies |  |
|  |  |  | *Hermetia* | *illucens* | black soldier fly |  |
|  |  |  | *Neopachygaster* |  | soldier flies |  |
|  |  |  | *Ptecticus* |  | soldier flies |  |
|  |  | Syrphidae |  |  | hover flies | 4 |
|  |  | Tabanidae |  |  | horse flies | 8 |
|  |  |  | *Tabanus* |  | horse flies |  |
|  |  |  | *Tabanus* | *fulvulus* | horse fly |  |
|  |  | Tachinidae |  |  | tachinid flies | 18 |
|  |  | Tephritidae |  |  | fruit flies | 6 |
|  |  |  | *Neotephritis* |  | fruit flies |  |
|  |  | Tipulidae s.l. |  |  | crane flies | 74 |
|  |  |  | *Dolichopeza* |  | crane flies |  |
|  |  |  | *Elephantomyia* | *westwoodi* | crane fly |  |
|  |  |  | *Epiphragma* |  | crane flies |  |
|  |  |  | *Epiphragma* | *solatrix* | crane fly |  |
|  |  |  | *Erioptera* |  | crane flies |  |
|  |  |  | *Geranomyia* |  | crane flies |  |
|  |  |  | *Gnophomyia* |  | crane flies |  |
|  |  |  | *Gnophomyia* | *trisstissima* | crane fly |  |
|  |  |  | *Limonia* |  | crane flies |  |
|  |  |  | *Nephrotoma* |  | tiger crane flies |  |
|  |  |  | *Rhipidia* |  | crane flies |  |
|  |  |  | *Rhipidia* | *domestica* | crane fly |  |
|  |  |  | *Tipula* |  | crane flies |  |
|  |  | Trichoceridae |  |  | winter crane flies | 20 |
|  |  |  | *Trichocera* |  | winter crane flies |  |
|  |  | Ulidiidae |  |  | picture-winged flies | 2 |
|  |  |  | *Delphinia* | *picta* | picture-winged fly |  |
|  |  | Xylomyidae |  |  | xylomyid flies | 2 |
|  |  |  | *Solva* |  | xylomyid flies |  |
| **Arachnida** |  |  |  |  | **arachnids** | **100** |
|  | **Araneae** |  |  |  | **spiders** | **100** |
|  |  | Agelenidae |  |  | grass & funnel weavers | 46 |
|  |  |  | *Agelenopsis* |  | grass & funnel weavers |  |
|  |  |  | *Agelenopsis* | *naevia* | grass & funnel weaver |  |
|  |  |  | *Barronopsis* |  | grass & funnel weavers |  |
|  |  |  | *Coras* |  | grass & funnel weavers |  |
|  |  | Anyphaenidae |  |  | ghost spiders | 30 |
|  |  |  | *Hibana* |  | ghost spiders |  |
|  |  | Araneidae |  |  | orb weavers | 18 |
|  |  |  | *Verrucosa* |  | orb weavers |  |
|  |  | Clubionidae |  |  | sac spiders | 10 |
|  |  |  | *Elaver* |  | sac spiders |  |
|  |  | Corinnidae |  |  | antmimics & ground sac spiders | 38 |
|  |  |  | *Castianeira* |  | antmimics & ground sac spiders |  |
|  |  |  | *Meriola* |  | antmimics & ground sac spiders |  |
|  |  |  | *Phrurotimpus* |  | antmimics & ground sac spiders |  |
|  |  |  | *Scotinella* |  | antmimics & ground sac spiders |  |
|  |  |  | *Trachelas* |  | antmimics & ground sac spiders |  |
|  |  | Ctenidae |  |  | wandering spiders | 2 |
|  |  | Dysderidae |  |  | woodlouse hunters | 6 |
|  |  |  | *Dysdera* | *crocata* | woodlouse hunter |  |
|  |  | Filistatidae |  |  | crevice spiders | 6 |
|  |  |  | *Kukulcania* | *hibernalis* | Southern house spider |  |
|  |  | Gnaphosidae |  |  | ground spiders | 48 |
|  |  |  | *Cesonia* |  | ground spiders |  |
|  |  |  | *Herpyllus* |  | ground spiders |  |
|  |  |  | *Herpyllus* | *ecclesiasticus* | Eastern parson spider |  |
|  |  | Linyphiidae |  |  | dwarf & sheetweb spiders | 22 |
|  |  | Lycosidae |  |  | wolf spiders | 40 |
|  |  | Mimetidae |  |  | pirate spiders | 4 |
|  |  |  | *Mimetus* |  | pirate spiders |  |
|  |  | Miturgidae |  |  | prowling spiders | 6 |
|  |  |  | *Strotarchus* |  | prowling spiders |  |
|  |  | Oecobiidae |  |  | wall spiders | 28 |
|  |  |  | *Oecobius* |  | wall spiders |  |
|  |  | Oonopidae |  |  | goblin spiders | 16 |
|  |  |  | *Orchestina* |  | goblin spiders |  |
|  |  | Oxyopidae |  |  | lynx spiders | 4 |
|  |  | Pholcidae |  |  | cellar spiders | 84 |
|  |  |  | *Pholcus* |  | cellar spiders |  |
|  |  |  | *Spermophora* | *senoculata* | cellar spider |  |
|  |  | Pisauridae |  |  | fishing & nursery web spiders | 4 |
|  |  |  | *Dolomedes* |  | fishing spiders |  |
|  |  | Salticidae |  |  | jumping spiders | 50 |
|  |  |  | *Metacyrba* |  | jumping spiders |  |
|  |  |  | *Phidippus* |  | jumping spiders |  |
|  |  | Scytodidae |  |  | spitting spiders | 16 |
|  |  |  | *Scytodes* | *thoracica* | spitting spider |  |
|  |  | Segestriidae |  |  | tube web spiders | 6 |
|  |  |  | *Ariadna* |  | tube web spiders |  |
|  |  |  | *Ariadna* | *bicolor* | tube web spider |  |
|  |  | Tetragnathidae |  |  | long-jawed spiders | 2 |
|  |  |  | *Leucauge* |  | orchard spiders |  |
|  |  | Theridiidae |  |  | cobweb spiders | 100 |
|  |  |  | *Coleosoma* |  | cobweb spiders |  |
|  |  |  | *Latrodectus* |  | widow spiders |  |
|  |  |  | *Parasteatoda* | *tepidariorum* | common house spiders |  |
|  |  |  | *Steatoda* |  | cobweb spiders |  |
|  |  | Thomisidae |  |  | crab spiders | 32 |
|  |  | Uloboridae |  |  | cribellate orb weavers | 2 |
|  |  |  | *Uloborus* |  | cribellate orb weavers |  |
|  | **Acari** |  |  |  | **mites & ticks** | **100** |
|  |  | Erythraeidae |  |  | long-legged velvet mites | 2 |
|  |  | Galumnidae |  |  | armored mites | 12 |
|  |  | Ixodidae |  |  | hard ticks | 18 |
|  |  |  | *Amblyomma* |  | hard ticks |  |
|  |  |  | *Amblyomma* | *americanum* | lonestar tick |  |
|  |  |  | *Dermacentor* |  | dog & wood ticks |  |
|  |  |  | *Dermacentor* | *variabilis* | American dog tick |  |
|  |  | UnID Oribatida |  |  | armored mites | 46 |
|  |  | Plateremaeidae |  |  | armored mites | 2 |
|  |  | Pyroglyphidae |  |  | dust mites | 76 |
|  |  |  | *Dermatophagoides* |  | house dust mites |  |
|  |  | Tetranychidae |  |  | spider mites | 6 |
|  |  |  | *Bryobia* |  | clover mites |  |
|  |  | Trombidiidae |  |  | velvet mites | 4 |
|  | **Opiliones** |  |  |  | **harvestmen & daddy-longlegs** | **16** |
|  |  | Phalangiidae |  |  | harvestmen & daddy-longlegs | 4 |
|  | **Pseudoscorpionida** |  |  |  | **pseudoscorpions** | **20** |
|  |  | Chernetidae |  |  | pseudoscorpions | 2 |
|  |  | Chthoniidae |  |  | pseudoscorpions | 4 |
| **Chilopoda** |  |  |  |  | **centipedes** | **42** |
|  | **Geophilomorpha** |  |  |  | **soil centipedes** | **8** |
|  | **Lithobiomorpha** |  |  |  | **stone centipedes** | **18** |
|  |  | Lithobiidae |  |  | stone centipedes | 14 |
|  | **Scolopendromorpha** |  |  |  | **bark centipedes** | **12** |
|  |  | Scolopendridae |  |  | bark centipedes | 12 |
|  |  |  | *Hemiscolopendra* | *marginata* | Florida blue centipede |  |
|  | **Scutigeromorpha** |  |  |  | **house centipedes** | **32** |
|  |  | Scutigeridae |  |  | house centipedes | 32 |
|  |  |  | *Scutigera* | *coleoptrata* | house centipede |  |
| **Diplopoda** |  |  |  |  | **millipedes** | **82** |
|  | **Callipodida** |  |  |  | **crested millipedes** | **12** |
|  |  | Abacionidae |  |  | crested millipedes | 12 |
|  |  |  | *Abacion* |  | crested millipedes |  |
|  | **Julida** |  |  |  | **millipedes** | **42** |
|  |  | Julidae |  |  | julid millipedes | 38 |
|  |  | Parajulidae |  |  | parajulid millipedes | 4 |
|  | **Polydesmida** |  |  |  | **flat-backed millipedes** | **72** |
|  |  | Paradoxosomatidae |  |  | flat-backed millipedes | 58 |
|  |  |  | *Oxidus* | *gracilis* | greenhouse millipede |  |
|  |  | Polydesmidae |  |  | flat-backed millipedes | 26 |
|  |  |  | *Polydesmus* |  | flat-backed millipedes |  |
|  |  |  | *Pseudopolydesmus* |  | flat-backed millipedes |  |
|  |  |  | *Scytonotus* |  | flat-backed millipedes |  |
|  |  | Xystodesmidae |  |  | flat-backed millipedes | 6 |
|  |  |  | *Apheloria* |  | flat-backed millipedes |  |
|  | **Spirobolida** |  |  |  | **round-backed millipedes** | **20** |
|  |  | Spirobolidae |  |  | round-backed millipedes | 18 |
|  |  |  | *Narceus* |  | round-backed millipedes |  |
|  |  |  | *Narceus* | *americanus* | round-backed millipede |  |
|  | **Spirostreptida** |  |  |  | **spirostreptid millipedes** | **2** |
|  |  | Cambalidae |  |  | spirostreptid millipedes | 2 |
|  |  |  | *Cambala* |  | spirostreptid millipedes |  |
| **Malacostraca** |  |  |  |  | **crustaceans** | **86** |
|  | **Decapoda** |  |  |  | **decapods** | **2** |
|  | **Isopoda** |  |  |  | **isopods** | **84** |
|  |  | Armadillidiidae |  |  | pillbugs & roly polies | 78 |
|  |  |  | *Armadillidium* | *nasatum* | pillbug or roly poly |  |
|  |  |  | *Armadillidium* | *vulgare* | pillbug or roly poly |  |
|  |  | Oniscidae |  |  | woodlice & sowbugs | 6 |
|  |  |  | *Oniscus* |  | woodlice & sowbugs |  |
|  |  | Porcellionidae |  |  | woodlice & sowbugs | 10 |
|  |  | Trachelipodidae |  |  | woodlice & sowbugs | 4 |
|  |  |  | *Trachelipus* |  | woodlice & sowbugs |  |
